# High frequency and long duration of toothbrushing can potentially reduce the risk of common systemic diseases in late adolescence

**DOI:** 10.1111/scd.12670

**Published:** 2021-10-22

**Authors:** Masanobu Abe, Akihisa Mitani, Liang Zong, Chun‐Dong Zhang, Kazuto Hoshi, Shintaro Yanagimoto

**Affiliations:** ^1^ Division for Health Service Promotion The University of Tokyo Tokyo Japan; ^2^ Department of Oral and Maxillofacial Surgery The University of Tokyo Hospital Tokyo Japan; ^3^ Division of Epigenomics National Cancer Center Research Institute Tokyo Japan; ^4^ Department of Gastrointestinal Surgery Graduate School of Medicine The University of Tokyo Tokyo Japan

Periodontal diseases are the most common oral diseases affecting the supporting structures of the teeth.^1^ They are not only responsible for tooth loss but also associated with various systemic diseases including noncommunicable diseases (NCDs) such as cardiovascular diseases, diabetes, and respiratory diseases.^2^ Most of the extant studies of the relationship between periodontal diseases and systemic diseases have targeted middle‐aged and elderly people. On the other hands, adolescents have not been focused on because severe periodontitis is quite rare in this life‐stage. In our recent survey (a retrospective review of the mandatory medical questionnaires administered as a part of legally required medical checkup between 2017 and 2019 in a University in Tokyo), 36.5% of 9098 University students aged 17–19 years were aware of gum bleeding when they brush their teeth. Importantly, multivariate regression analysis showed that the awareness of gum bleeding was closely associated with the histories of asthma/cough‐variant asthma and otitis media/external with odds ratio (OR) 1.691 (95% confidence interval [CI]: 1.193–2.396) and 1.303 (1.091–1.556), respectively.^3^ These findings suggest the importance of periodontal healthcare from the early stage of life.

Infrequent toothbrushing has been suggested to be associated with severe forms of periodontal disease since many decades.^4^, ^5^, ^6^, ^7^ However, a systematic review could not show sufficient evidence to justify the association because there were few studies evaluating the issue in detail.^8^, ^9^ Recently, we have focused on oral hygiene behavior as a factor that may be concerned to gingival health status in late adolescents. We have surveyed frequency and duration of toothbrushing of the 9098 University students aged 17–19 and addressed their association with awareness of gum bleeding. For the frequency of toothbrushing a day, “twice” was the most common frequency (65.8%) followed by “one time or less” (20.6%) and “three times or more” (13.6%). In a multivariate regression analysis, for students who brushed their teeth “one time or less,” the risk of gum bleeding was 2.36 (95% CI: 2.02–2.76) times higher than that for those who brushed their teeth “three times or more”. For students who brushed their teeth “twice,” the risk of gum bleeding was 1.45 (1.27–1.67) times higher than that for those who brushed their teeth “three times or more”.

For the duration of toothbrushing each time, “2–3 min” was the most common brushing duration (47.3%) followed by “4 min or more” (36.1%) and “1 min or less” (16.6%). In a multivariate regression analysis, for students who brushed their teeth for “1 min or less,” the risk was 1.57 (95% CI: 1.39–1.78) times that for those who brushed them for “4 min or more”. For students who brushed their teeth for “2–3 min,” the risk was 1.26 (1.14–1.39) times that for those who brushed them for “4 min or more”. In addition, male sex was found to be a risk factor of gum bleeding [1.29 (1.15–1.44) times greater risk than females]. We found that the risk of gum bleeding was increased with lower frequency and shorter duration of toothbrushing (Figure 1).^10^ In other words, the risk of periodontal diseases decreases as the frequency/duration of tooth brushing increases. From the perspective of preventing periodontal disease, brushing “three times or more” a day, “4 min or more” each time would be recommended.

**FIGURE 1:**
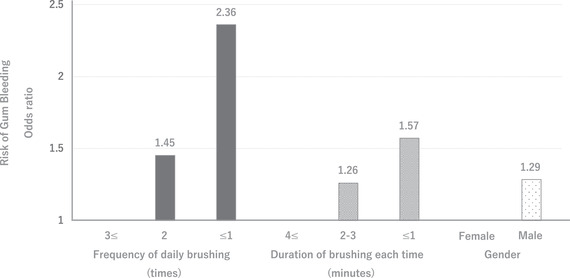
The risk of gum bleeding increases as the frequency/duration of toothbrushing decreases

Although there are no government‐sponsored programs to promote dental care among late adolescents in Japan,^11^ our findings strongly suggest the necessity and importance of increasing oral health‐consciousness in younger generations. Encouraging toothbrushing in younger generations may not only prevent severe periodontitis but also improve systemic health status which would be affected by periodontal diseases.

## CONFLICTS OF INTEREST

The authors declare no conflict of interest.
